# Chemically Activated Carbon Based on Biomass for Adsorption of Fe(III) and Mn(II) Ions from Aqueous Solution

**DOI:** 10.3390/ma16031251

**Published:** 2023-02-01

**Authors:** Amal M. Elewa, Ahmed A. Amer, Mohamed F. Attallah, Hamdy A. Gad, Zehbah Ali Mohamed Al-Ahmed, Inas A. Ahmed

**Affiliations:** 1 Department of Chemistry, Faculty of Science, Zagazig University, Zagazig 44519, Egypt; 2 Analytical Chemistry and Control Department, Hot Laboratories and Waste Management Center, Atomic Energy Authority of Egypt, Cairo 13759, Egypt; 3 Department of Chemistry, Faculty of Science and Art, King Khalid University, Dhahran Aljanoub, Abha 62224, Saudi Arabia; 4 Department of Chemistry, Faculty of Science, King Khalid University, Abha 62224, Saudi Arabia

**Keywords:** activated carbon (AC), rice husk (RH), activation, heavy metals, adsorption, kinetics

## Abstract

Rice husk was converted into activated carbon (AC) as a byproduct of agricultural waste in an electric furnace at 700 °C and chemically activated using three distinct processes: NaOH AC(C), acetic acid AC(C-1), phosphoric acid AC(C-2), and carbonization AC(C-3) without any chemical activation. To characterize the activated carbon and the removal efficiencies of Fe(III) and Mn(II) from aqueous solutions, various analytical tools were used. The results revealed that the capacities of the four adsorbents to adsorb Fe(III) or Mn(II) from an aqueous solution differ significantly. AC(C-3) was chosen for additional research. The impact of different operational factors, including pH, contact time, adsorbent dosage, starting metal ion concentration, interfering ions, and temperature, were investigated. The optimum pH values for Fe(III) and Mn(II) adsorption were found to be pH 3 and pH 6, respectively. The results obtained were utilized to assess the kinetics and thermodynamics of the adsorption process. The sorption of Fe(III) and Mn(II) ions was found to be a pseudo-second-order kinetic process, and the equilibrium data were fitted with the Langmuir isotherm. Additionally, the evidence suggests that an endothermic mechanism governs the adsorption process. The maximum adsorption capacities of Fe(III) and Mn(II) were 28.9 and 73.47 mg/g, respectively.

## 1. Introduction

The term “activated carbon” (AC) describes highly carbonaceous substances made from wood, coal, coconut shells, cones, etc., that have a high porosity and sorption capacity. One of the regularly employed adsorbents for the elimination of several contaminants from water and air bodies is AC [1,2]. Since AC is produced using agricultural and waste materials, it has proven to be a great substitute for previously used, expensive, nonrenewable sources. The use of activated carbon to remove harmful impurities, whether in the treatment of water or air, has become essential [3]. There are two ways to activate coal, one physical and one chemical. The various activation processes are, in the great majority, variations of a basic procedure which is the carbonization or pyrolysis of the raw material. There are also three basic physical forms of activated carbon: powdered (powder), granulated, and pelletized. Thus, the characteristics of activated carbon depend on the raw material used (vegetable or mineral) and the activation process (chemical or physical). As a result, each process will have different adsorption properties and different uses [3]. Despite being an ideal material for the removal of contaminants, the use of activated carbon is limited due to its high production cost, which has instigated new research with more viable options for reducing production costs. One of these options would be through the use of biomass as an alternative precursor material to conventional methods of producing activated carbon that currently uses mineral coal [4,5,6,7].

The world’s environmental pollution situation has worsened over the past century as a result of the acceleration of industrialization, which has increased the demand for indiscriminate resource extraction [8]. Contrary to other pollutants that can be seen in the environment, such as petroleum hydrocarbons and household and municipal garbage, trace metals can begin to accumulate to harmful levels without being observed. Metallic elements with a density greater than water are referred to as “heavy metals” [9]. Based on the idea that toxicity and heaviness are related, heavy metals also include metalloids like arsenic, which can be dangerous at low exposure levels. In recent years, environmental contamination by these metals has become a rising concern for both the environment and global health. Furthermore, human contamination has increased drastically due to an exponential expansion in their use in various industrial, agricultural, residential, and technical applications [10,11,12].

Iron and manganese (particularly the latter) generate distinct issues that have a variety of underlying reasons. Water pollutants that have exhibited greater removal efficiency by AC include but are not limited to heavy metals, pharmaceuticals, pesticides, natural organic matter, disinfection products, and micro-plastics. Granular activated carbon (GAC) is mostly used in aqueous solutions and adsorption columns for water treatment. Commercial AC is not only costly but also obtained from non-renewable sources [13,14,15].

This work aims to provide the methodologies used in the studies of activated carbon in the treatment of water for human consumption. The activated carbon produced from rice husk was chemically activated using three different methods in the current study: activation by NaOH (AC(C)), activation by acetic acid (AC(C-2)), activation by phosphoric acid (AC(C-1)), and the fourth sample by carbonization (AC(C-3). To characterize the activated carbon, Fe(III), and Mn(II) removal efficiencies from aqueous solutions, various analytical tools were used. The impact of different operational parameters, including pH, contact time, adsorbent dose, initial metal ion concentration, interfering ions, and temperature, was examined.

## 2. Experimental Procedures

### 2.1. Materials

Rice husk (RH) is a solid agricultural waste produced during the manufacture of rice and was sourced from local Egyptian farms, while the aqueous solutions of Fe(III) and Mn(II) were made from ferric and manganese chlorides by dissolving in deionized water.

### 2.2. Instruments

The pH values of different solutions were measured using a digital pH meter, [Thermo Scientific USA instruments]. The concentrations of metal ions were measured using atomic absorption (AA), [Varian AA240FS, Palo Alto, Austria]. Shaking of samples was performed using an orbital shaker [Thermo Scientific, Ireland, UK]. The surface area and pore characteristics of various samples were determined via nitrogen adsorption/desorption isotherms that were measured at 77 K on an automatic adsorption instrument [Quantachrome Instruments, Model Nova 1000 e series, USA] and data were calculated using NOVA Win 2.0 software. Elemental analysis was carried out by energy dispersive X-ray (EDX) [JMS-PLASMA-X2- with Resolving Power 1200] (JEOL, Akishima Tokyo, Japan). Fourier transform-infrared spectroscopy (FTIR) was recorded using a Mattson 5000 FTIR spectrometer. Scanning electron microscopy (SEM) of selected materials was carried out using a JSM T 20 JOEL, JAPAN apparatus in the secondary-electron image mode, at an accelerated voltage of 20 Kev.3.2.

### 2.3. Preparation of Activated Carbon

We performed post-treatment on rice husk materials that had been collected from the rice factory and washed with deionized water to remove any particles before drying in a 100 °C oven for 2 h. Approximately 40 g of RH was mixed and stirred with 100 mL of 1 M NaOH for 90 min before heating at 100 °C. The sample was calcined in an electric furnace at 700 °C for 90 min (heating rate: 50 °C per 10 min). The sample was cooled and ground to create powdered activated carbon at room temperature, followed by washing with hot de-ionized water for 15 min. The washing process was repeated four times, followed by drying in the oven at 100 °C for 120 min to record the sample AC. Finally, it was cooled to room temperature, and the sample weight was determined. The same procedure was used to activate RH with 1M CH_3_COOH and 1M H_3_PO_4_ to record the sample AC(C-1) and AC(C-2). The sample AC(C-3) was only fired in the electric furnace at 700 °C for 90 min (heating rate: 50 °C/10 min) after post-treatment without any activation. The various types of the prepared activated carbon are recorded in Table 1.

### 2.4. Batch Sorption Experiments

The batch equilibrium approach was used to carry out the adsorption studies.

- The effect of pH on metal adsorption was investigated. In this case, 0.05 g of activated carbon was incubated for 120 min at 25 °C with 25 mL of Fe(III) or Mn(II) ions. An amount of 0.1N NaOH or 0.1N HCl was used to adjust the pH of this combination to a range of 1 to 6. The residual concentration of each element was calculated by atomic absorption after the mixture had been filtered using Whatman filter paper and agitated until equilibrium. Adsorbent dosages ranging from 0.025 to 0.5 g were added to various bottles containing 25 mL of metal solution, and the mixture was vigorously shaken for 2 h at a speed of 100 rpm, a temperature of 25 °C, and at pH 3 for Fe(III) and pH 6 for Mn(II). Then, each flask’s contents were filtered and examined.
- Different time intervals, including 5, 15, 30, 60, 120, 180 min., and 24 h, were tested to determine the impact of contact time on the elimination of metal ions. The activated carbon (0.05 g) adsorbent was applied to various conical flasks holding 25 mL of metal ions at a temperature of 25 °C and at pH 3 for iron and pH 6 for manganese solutions. For each of the various selected contact times, the bottles were closed, set on a mechanical shaker, and agitated at 100 rotations per minute (rpm). Each agitation period was followed by filtering and analysis of the contents of each flask.
- Temperature-dependent rates and degrees of adsorption were studied for 120 min at 25, 35, 50, and 65 °C at pH 3 for Fe(III) and pH 6 for Mn(II). The removal of Fe(III) and Mn(II) ions from aqueous solution was investigated using 0.05 g of adsorbent at pH 3 for Fe(III) and pH 6 for Mn(II). The time to reach equilibrium was 1 h.
- How some interfering ions, such as anions or cations, affected the effectiveness of adsorption was investigated. The percentage of Fe(III) and Mn(II) uptake in various cations, such as Na(I), Mg(II), and K(I), as well as other anions, such as chlorides, sulfates, and nitrates, was studied. To address this issue, 0.03 g of adsorbent was agitated with 10 mL of Fe(III) or Mn(II) starting solution containing the same concentration of interfering ion. After filtering, it was determined whether Fe(III) or Mn(II) ions were taken up. Adsorption uptake and the removal percentage were calculated using Equations (1) and (2):

(1) $$\mathrm{Adsorption}\;\mathrm{capacity}\;q_{e}=\frac{\left(C_{0}-C_{e}\right)V}{M}$$

(2) $$\mathrm{Removal}\;\mathrm{efficiency}\;\%=\frac{\left(C_{0}-C_{e}\right)}{C_{0}}\times 100$$

where q_e_ is the uptake of adsorbate per unit mass of adsorbent (mg/g),

C_o_ is the initial concentration of Fe(III) or Mn(II) in the aqueous solution (mg/L),

C_e_ is the final equilibrium concentration of test solution (mg/L),

M is the mass of adsorbent (g),

V is the volume of sample (L).

## 3. Results and Discussion

**Figure 1:**
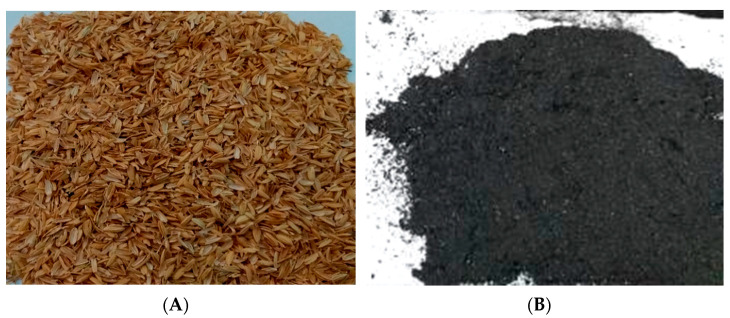
Photographic pictures for (**A**) raw rice husk (RH) at 25 °C, (**B**) rice husk (RH) AC carbonized at 700 °C (AC(C-3)).

The purpose of the current study was to determine whether it is feasible to treat waste water to remove dissolved ions, specifically Fe(III) and Mn(II), using activated carbons prepared from rice husk (RH). Photographic pictures for (A) raw rice husk (RH) at 25 °C, (B) rice husk (RH) AC carbonized at 700 °C (AC(C-3) are shown in Figure 1.

### 3.1. Adsorption of Metal Ions from Aqueous Solutions Using Prepared Rice Husk-Based Activated Carbons

The adsorption from solutions was investigated to test the prospective use of these carbons as universal adsorbents due to the potential practical applications of manufactured activated carbons. To show the activated carbon’s appropriateness for treating particular fluids and pollutants (such as organics, dyestuffs, metals, etc.), standard testing procedures are typically used and indicated in producer certificates [15]. For this purpose, Fe(III) and Mn(II) isotherm measurements are used in batch adsorption.

#### Preliminary Sorption Investigation

The main goals of this section were to investigate and select the best adsorbents to use for removing Fe(III) or Mn(II) from an aqueous solution. In this case, four different adsorbent samples prepared as previously described were tested for their ability to bind Fe(III) and Mn(II) ions. In this experiment, 0.05 g of each adsorbent was stirred for 2 h with 25 mL of metal ion solution containing 50 ppm of either Fe(III) or Mn(II), with pH levels ranging from pH 1.5 to 6. The findings in this regard are displayed in Table 2. According to the results of Table 2, the capacity of the four adsorbents to adsorb Fe(III) or Mn(II) from an aqueous solution varies significantly. Large variations in the absorption of both metal ions have been reported, pointing to various processes of interaction between the adsorbent surface and metal-containing species in the aqueous solution. In terms of Mn(II) elimination, AC (C-3) was shown to be superior. To carry out further research, AC (C-3) was chosen. Additionally, we examined the physical and chemical properties of AC (C-3).

### 3.2. Characterization of Prepared Activated Carbon

#### 3.2.1. Physical Properties

The Physical properties of selected prepared activated carbon samples under investigation were studied in terms of apparent density, packed density, elemental analyses, and carbon yield.

##### Apparent Density

The mass of the carbon atoms per unit volume, including the pores and inter particle gaps, is used to describe the apparent density of a solid. Table 3 makes it clear that generally speaking, rice husk treated with phosphoric acid exhibits an apparent density that is higher than the original Bagasse pulp, which may be connected to the presence of residual phosphorus [16]. The original rice husk’s perceived density is far lower than that of the unit. This implies that rice husk will float on the water’s surface when used to treat water. Even though the treated rice husk’s apparent density is higher than the untreated rice husk.

##### Packed Density

A tamping process described by [17] was used to measure the packed density. As can be seen from Table 3, the method used usually results in packed densities that are higher than the apparent densities. In the packed density, the solid material is compressed by repeatedly tapping on the glass cylinder, which causes the porous fluffy carbonaceous material to break down. As a result, the high values of the packed density contrast with the raw, hard particles that are devoid of any appreciable porosity. The existence of residual phosphorus could explain why AC(C-3) apparent density and packed density were 0.477 and 0.591g/cm^3^, respectively [18].

##### Yield

Yield is the quantity of the initial precursor that is still present after activation. The yields of the chosen produced activated carbons are displayed in Table 3.

##### BET Adsorption–Desorption Measurements

**Figure 2:**
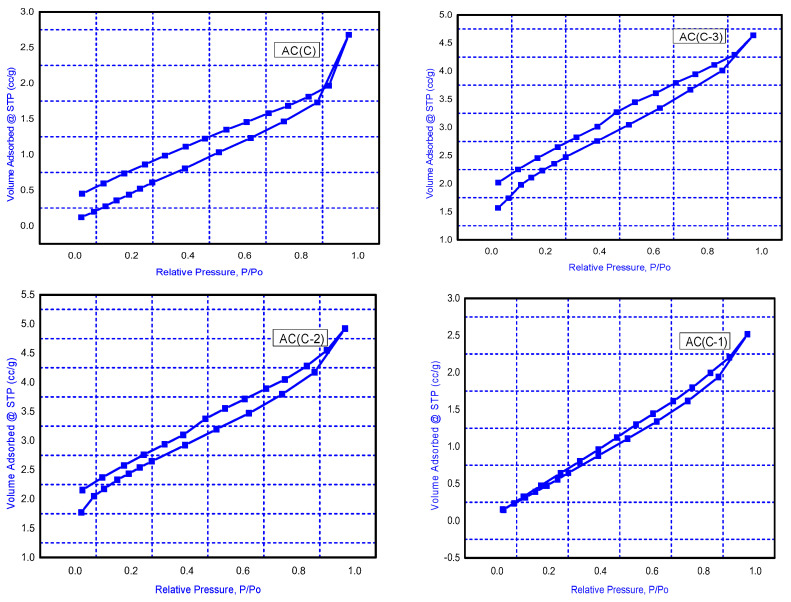
N_2_ Adsorption–desorption isotherm of the prepared activated carbons AC(C-3), AC(C-2), AC(C-2), and AC(C).

Figure 2 illustrates the N_2_ adsorption–desorption isotherms from type IV, the pore size distribution evidence from the meso-porous nature of the active carbon. Surface area, average pore radius, and total pore volume of the chosen produced activated carbons are displayed in Table 3 [19].

From the physical properties of the selected prepared activated carbon samples in Table 3, it is obvious that AC(C-3) has the highest surface area 228.46 (m^2^/g) and pore volume 0.0210 cc/g.

##### Elemental Analyses

**Figure 3:**
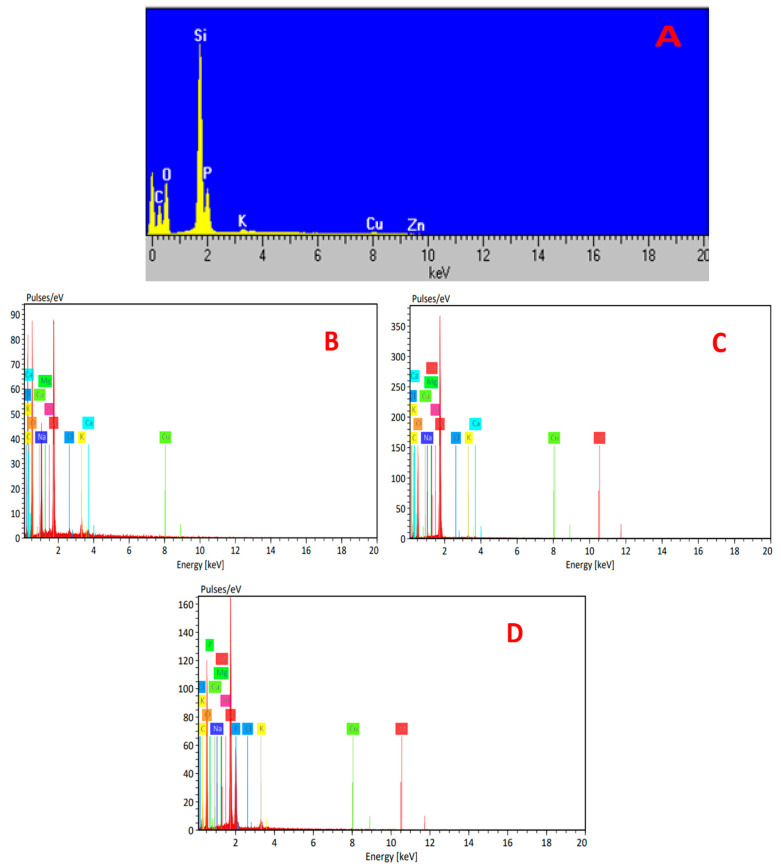
Elemental analyses by EDX spectrum of (**A**) AC(C-3), (**B**) AC(C), (**C**) AC(C-2), and (**D**) AC(C-1).

The energy dispersive X-ray (EDX) result for the AC(C-3) before metal ions adsorption is shown in Figure 2. According to the results in Table 4, the presence of carbon (36.12 wt%), oxygen (33.51 wt%), silicon (16.46 wt%), phosphorous (7.99 wt%), copper (2.96 wt%), zinc (2.44 wt%), and potassium (0.52 wt%) was confirmed in the AC(C-3) sample shown in Figure 3.

#### 3.2.2. Chemical Properties

##### Surface Functional Groups of the Prepared Adsorbents (FT-IR Analysis)

In their chemical composition, agricultural wastes mostly consist of cellulose, lignin, and hemicellulose [20]. One of the main components of cellulose is a polysaccharide made of glucose units. According to the AC(C-3) spectrum, the adsorbent has functional groups that could serve as proton donors, which is critical for coordination with heavy metals. The hydroxyl groups are attributed to the functional group indicated by the bands at 3400 cm^−1^. The C–O band and the C–H aliphatic band usually appear at 1100 cm^−1^ and 2900 cm^−1^, respectively [20]. Hemicellulose has comparable peaks to cellulose because they have a structural similarity, with the exception that glucose is not the only sugar present [21]. Lignin is the only component of the waste that contains aromatic species, albeit its structure has not yet been established. These aromatic components can produce peaks in a range of 1400–1600 cm^−1^. There might also be a carbonyl group, which produces a signal in the range of 1600–1800 cm^−1^ [22].

**Figure 4:**
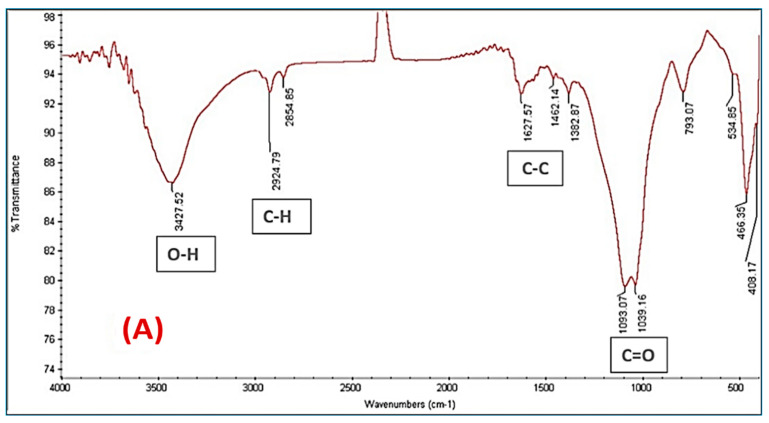

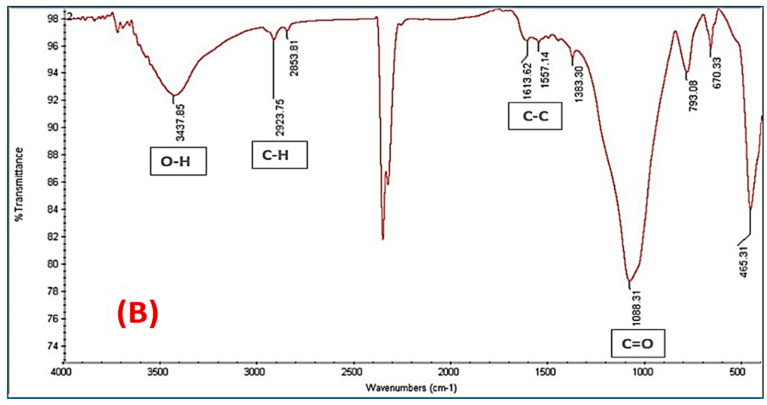
FTIR spectrum of AC(C-3): (**A**) AC(C-3) before adsorption, (**B**) AC(C-3) after adsorption Fe(III) and Mn(II).

Figure 4A depicts the predicted peaks that represent the active carbons before the adsorption of Fe(III) and Mn (II). At 3424 cm^−1^, the anticipated hydroxyl group peak may be seen. At 2921 and 1093 cm^−1^, respectively, are the aliphatic C–H peak and the C–O peak. The ortho-substituted group cyanate CNO at 2278 cm^−1^ and the C=C and C–C typical for the benzene ring appear at 1563 and 1511 cm^−1^ for C=C and 1454 and 1380 cm^−1^ for C–C, respectively. Figure 4B shows the active carbons following interaction with Fe(III) and (II), which reflect the adsorption of Fe(III) and Mn (II). Similar to this, the intensity level of the peaks, which reflect the active carbons before the adsorption of Fe(III) and Mn(II), is still low but has moved somewhat in position.

##### Scanning Electron Microscopy (SEM)

**Figure 5:**
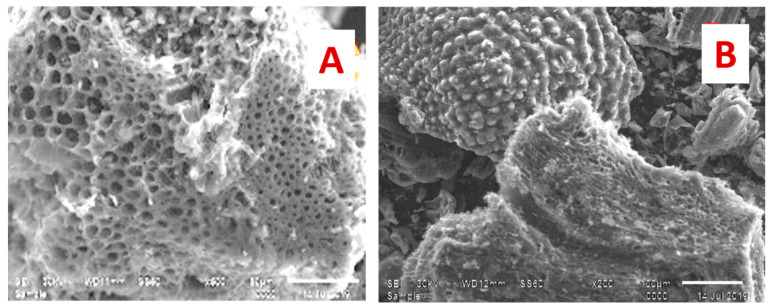
SEM of (AC(C-3) (**A**) before adsorption, (**B**) after adsorption Fe(III) and Mn(II).

Scanning electron microscopy (SEM) was carried out to observe the changes in the morphology of adsorbents after adsorption. The SEM images of AC were carried out using the JSM T 20 JOEL, JAPAN apparatus in the secondary-electron image mode, at an accelerated voltage of 20 Kev. Figure 5A illustrates a close view of AC before adsorption which shows a porous surface with regularly wide channels. The surface morphology of the adsorbent after the adsorption of metal ions covered with Fe(III) and (II) ions is shown in Figure 5B.

### 3.3. Iron and Manganese Adsorption Studies

#### 3.3.1. Effect of pH on the Adsorption of Iron and Manganese Ions

**Figure 6:**
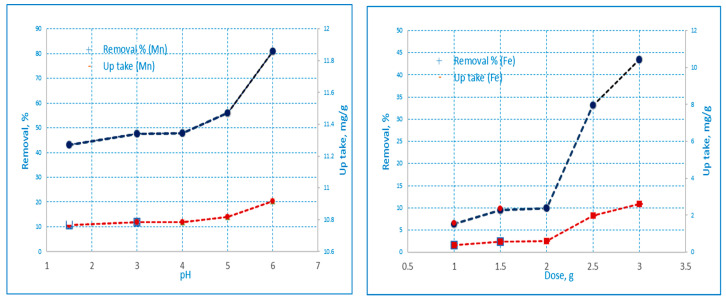
The influence of pH on the absorption and removal by AC(C-3) of Fe(III) and Mn(II) from aqueous solutions (conc. = 50 mg/L, time = 2 h, wt. = 0.05 gm, agitation velocity = 100 rpm and temp. 25 °C) by percentage.

The pH value reflects the concentration level of H^+^ in water; the ion exchange and electrostatic interaction are greatly influenced by H^+^ concentration [23]. The effect of pH value on the adsorption capacity of heavy metals is one of the important parameters for the sorption process that significantly influences adsorption characteristics, the ionization and protonation degree of the adsorbent, and the surface charges [24]. Metal ion adsorption increases with an increase in the pH value (below the precipitate limits). The effect of pH on the adsorption of Fe(III) and Mn(II) ions onto AC(C-3) samples, respectively, from aqueous solutions was determined. The solution pH was varied from 1 to 6 as shown in Figure 6. It was observed that the adsorption of Fe(III) and Mn(II) increased as the pH of the solution increased for the biosorbents under investigation. For Fe(III), the uptake of metal ions decreased at lower pH due to the high concentration of H^+^ that was absorbed on the carbon surface and rendered it positively charged. The amount of adsorbed iron on the AC increased significantly from pH 1 to 3 as shown in Table 5 [25]. On the other hand, when the pH value was high, Fe (II) was prone to hydrolysis reactions forming iron hydroxide precipitation that could spread easily into the inner porosity of the ACs, precipitate in the macropores, and cover the outer surface of the carbon particles [26].

For Mn(II), this occurs for the Mn^+2^ (H_2_O)_6_ ion in aqueous solution, which interacts with a negatively charged adsorbent. At lower pH, adsorption of Mn(II) was low due to an increase in positive charge at the active adsorbent sites. The concentration of H^+^ ions is high at low pH, so there is rivalry between H^+^ ions and Mn^+2^ (H_2_O)_6_ ions in the bulk of the solution to bind with the adsorbent surface that is negatively charged. The adsorption of Mn(II) increased as the solution pH increased to a value of pH 6, as shown in Table 5. At pH > 6, Mn(II) adsorption decreases due to the precipitation of Mn(II) hydroxide ions and the formation of manganese hydroxide precipitate [27]. 

#### 3.3.2. Effect of Contact Time on Adsorption of Iron and Manganese

**Figure 7:**
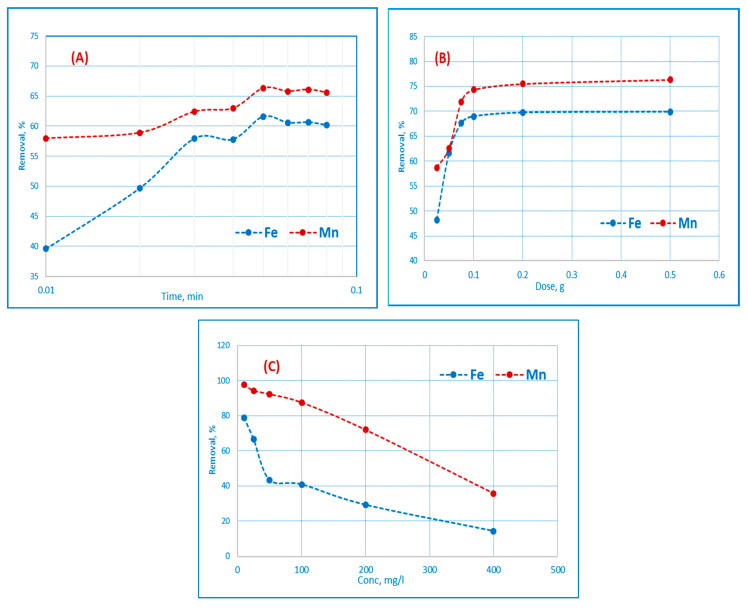
Effect of (**A**) adsorbent dosage, (**B**) concentration, and (**C**) contact time on Fe(III) and Mn(II) percentage removal by AC(C-3) (time = 60 min, pH = 3 and 6 respectively for Fe(III) and Mn(II), agitation velocity = 100 rpm and temp = 25 °C).

The time of contact is critical for efficient adsorbate–adsorbent bonding. Table 6 illustrates that the rate of adsorption significantly increased to 15.39 and 15.57 mg/g, respectively, for the metal ions present for 60 min of contact time. Shown in Figure 7A, the rate of removal of metal ions increasing with the increase of adsorption time could be due to greater contact between the surface of the adsorbent and metal ions. The increase in the rate of adsorption is due to the existence of a large number of active surface sites of active carbon and the rapid diffusion of metal ions from the bulk of the solution to the adsorbent surface [28]. However, there is no major improvement in the removal of metal ions due to further increases in contact time (until equilibrium); this is because it is simply due to the saturation of the active site which does not enable further adsorption. 

#### 3.3.3. Effect of Adsorbent Dose

The adsorbent dosage determines the adsorbent’s capacity for a particular initial concentration of a metal solution, making it a crucial parameter in adsorption research. Table 7 and Figure 7B illustrate how the amount of adsorbent used affects the uptake (mg/g) and percentage removal of Fe(III) and Mn(II) on AC(C-3). The results demonstrate that as the amount of activated carbon was raised, the percentage of Fe(III) and Mn(II) adsorption initially rose, where the availability of more active metal ion absorption sites at higher adsorbent dose levels may be the cause of the increased metal ion adsorption with increasing activated carbon concentrations [29]. Because the adsorbent is already adsorbing the available metal ions, subsequent increases in the adsorbent amount had no impact on the metal adsorption [30].

The metal ions uptake per unit mass decreased with the increase in adsorbent dosage for both adsorbents. The reason for this trend may be attributed to the fact that at high sorbent dosages, the available metal ions are not able to cover all the exchangeable sites on the biosorbent, resulting in low metal ions uptake [31]. 

#### 3.3.4. Effect of Initial Metal Ion Concentration

The effect of the initial metal ion concentration on Fe(III) and Mn(II) was investigated at 10, 25, 50, 100, 200, and 400 ppm. The pH values for Fe(III) and Mn(II) were set at pH 3 and pH 6, respectively. Figure 7C and Table 8 show the uptake of Fe(III) and Mn(II) ions on AC (C-3). At 25 °C, increasing the concentration of metal ions increased the absorption of Fe(III) from 3.95 to 29.25 mg/g and Mn(II) from 4.89 to 71.99 mg/g, respectively. The equilibrium uptake increased until saturation as the initial concentration increased. This shows that when there are more suitable sites involved early and the number of available sites is significantly greater than the number of metal ions species that can be adsorbed, the adsorption process appears to progress quickly. High-affinity sites start to become saturated as the metal concentration rises, and energetically unfavorable sites (low-affinity surface spots) start to participate in the adsorption process, which reduces uptake [32].

#### 3.3.5. Effect of Interfering Ions (Cations and Anions)

The adsorption process of heavy metal ions can be inhibited by the presence of coexisting ions. Table 9 displays the ability of the coexisting anions and cations to bind to AC(C-3). These findings demonstrate that optimal values refer to the ions and cations that have the biggest effects on the capacity of absorption. In addition to various anions like Cl^−^, SO_4_^2−^, and NO_3_^−^ (manganese and ferric salts), the influence of some cations like Na^+^, Mg^2+^, and K^+^ (chloride form) on the absorption of Fe(III) and Mn(II) was also investigated under ideal conditions as in Table 9. It demonstrated the uptake of Fe(III) and Mn(II) ions on AC(C-3), showing that the percentage of Fe(III) removal was reduced by 20.08 percent in the presence of cations Na^+^, Mg^2+^, and K^+^ while the percentage of Fe(III) removal was reduced by 30.48% in the case of anions Cl^−^, SO_4_^2−^, and NO_3_^−^. However, the percentage of Mn(II) removal was reduced by 12.02%. This suggests that the potential for AC to sorb and the potential for Fe(III) and Mn(II) to eliminate are both decreased by the presence of anions or cations, respectively. The presence of other ions in the solution that might compete with the metal ion of interest for sorption sites may be the cause of the decrease in sorption potential in the presence of cations. The binding of this metal ion is then lessened. The frequency of the various ions’ biomass binding determines how much inhibition occurs. However, the reduction in sorption potential in the presence of anions may be due to the formation of complexes with metal ions that have a higher solution affinity than free metal ions, or by inhibition of the sorption of the investigated metal ions [33].

### 3.4. Adsorption Isotherms

The volume of adsorbate adsorbed at a constant temperature by the unit mass of the adsorbent from the liquid process is shown on the adsorption isotherm. For the best possible adsorption device design, the analysis of adsorption equilibrium data is crucial. The adsorption isotherm is a relationship between the concentration of metal ions in the solution and the number of metal ions adsorbed onto the adsorbents. Numerous isothermal models have been extensively utilized to analyze the adsorption data. In this study, Freundlich, Langmuir and Temkin adsorption models were used to analyze how Mn(II) and Fe(III) ions interacted with adsorbents [34].

#### 3.4.1. Langmuir Isotherm

The linearized form of the Langmuir equation is given by Equation (3) [35].

RL = 1/(1+ bC_o_) (3)

C_e_/q_e_ = (1/q_m_b) + (C_e_/q_m_) (4)

**Figure 8:**
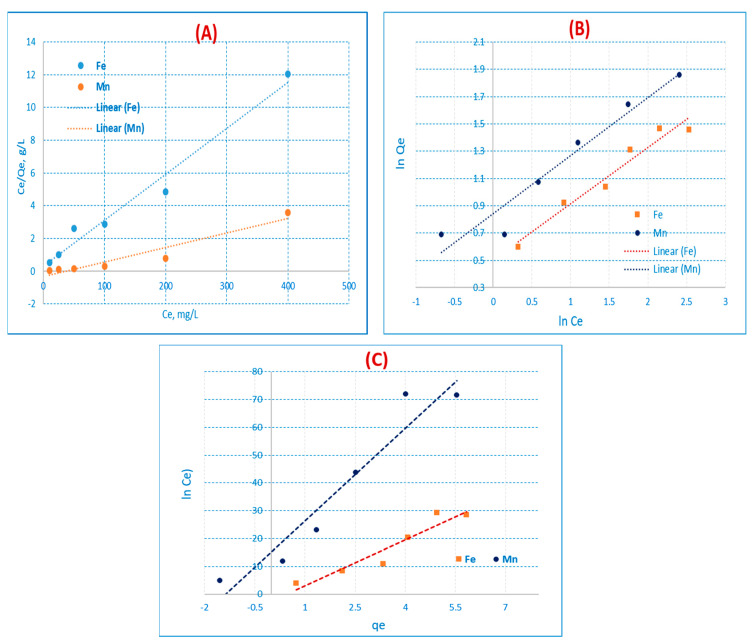
Langmuir (**A**), Freundlich (**B**), Temkin (**C**) isotherm plots for the adsorption of Fe(III) and Mn(II) using AC(C−3).

If C_e_ is the solution equilibrium adsorbate concentration (mg/L), then the adsorbent equilibrium concentration (mg/g), q_m_ is the adsorbent monolayer power (mg/g), b is the adsorbent constant (L/mg), etc. According to Equation (3), a plot of C_e_/q_e_ versus Ce should be a straight line with a slope of 1/qm and an intercept of (1/q_m_) b, as depicted in Figure 8A. Fe(III) and Mn(II) ions sorbed onto AC(C-3) are depicted as a straight line in the graph of (Ce/q_m_) vs Ce (Figure 8A). The slope and intercept of the plot were used to calculate the numerical values of the q_max_ and b constants. The amount of the monolayer covered by the q_max_ adsorption power of the monolayer defines the total capacity of the adsorbent for a certain metal ion. The phrase separation factor, also known as the equilibrium constant RL, has been used to characterize the basic properties of the Langmuir isotherm and is found in Equation (4). The R_L_ value reveals the adsorption type according to Table 10 [36].

For AC(C-3), the Langmuir model determined the adsorption potential q_max_ to be 29.2 and 71.9 (mg/g) for Fe^3+^ and Mn^2+^, respectively. The positive adsorptions of Fe^3+^ and Mn^2+^ for AC(C-3) were confirmed in the current investigation by R_L_ values of 0.11 and 0.028, respectively.

#### 3.4.2. Freundlich Isotherm

Equations (5) and (6) both represent the traditional Freundlich isotherm [37] in logarithmic form.

q_e_ = K_F_ Ce^1/n^ (5)

log q_e_ = log K_F_ + 1/𝑛 log Ce (6)

where K_F_ (mg/g) and n are the adsorption strength and power constants, respectively. The K_F_ values can be utilized to reflect the relative potential of adsorption, and the value of n denotes either preferential or unfavorable adsorption (*n* > 1) [38]. Analysis of the Freundlich sorption isotherm applicability was performed by graphing log q_e_vs log C_e_ as in Figure 8B. The intercept and slope of the linear regressions in Table 10 were used to estimate the K_F_ and 1/n values, respectively. 

#### 3.4.3. Temkin Isotherm

Temkin and Pyzhey [38] considered the effects of several indirect adsorbate or adsorbate interactions on adsorption isotherms and suggested that because of these interactions the heat of adsorption of all molecules in the layer would decrease linearly with coverage. The Temkin equation is given by Equation (7).

q_e_ = B_T_ ln A_T_ + B_T_ ln C_e_ (7)

where, B_T_ = RT/b_T_, T (K) is the absolute temperature; R is the universal gas constant (8.314 J/K mol); A_T_ (L/mg) is the Temkin isotherm equilibrium binding constant that corresponds to the maximum binding energy; B_T_ is related to the heat of adsorption; b_T_ is the Temkin isotherm constant; q_e_ and C_e_ are the amount of adsorbate adsorbed per unit weight of adsorbent and equilibrium concentration of adsorbate remaining in solution, respectively. The Temkin sorption isotherm was analyzed by plotting ln C_e_ against q_e_ as shown in Figure 8C and the values of B and A are determined from the intercept and slope of the linear regressions and summarized in Table 10.

### 3.5. Adsorption Kinetic Studies

#### 3.5.1. Kinetic First-Order Model

**Figure 9:**
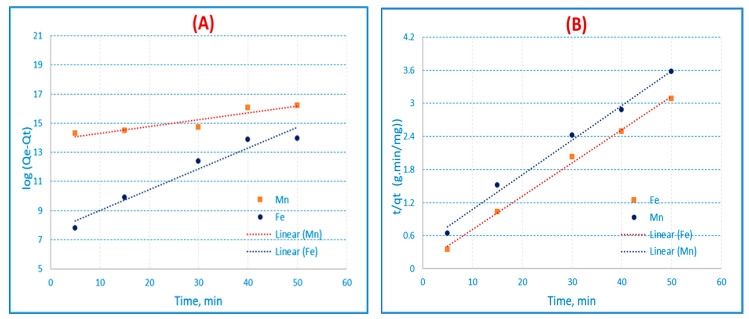
Kinetic (**A**) pseudo-first order and (**B**) pseudo-second-order on AC(C-3) for Fe(III) and Mn(II) respectively (conc. = 50 mg/L, t = 60 min, shaker = 100 rpm, and pH = 3 for Fe(III), and pH = 6 for Mn(II) at 25 °C).

Pseudo-first and pseudo-second-order kinetic models were applied to the experimental data in order to assess the adsorption kinetics [39,40]. One of the most often employed equations for liquids is the Lagergren first order rate equation. Equation (8) expresses the adsorption studies:

Log (q_e_ − q_t_) = log q_e_ − k_f_ t/2.303 (8)

where the amounts of solute adsorbed (mg/g) at equilibrium and at time t are, respectively, q_e_ and q_t_ (min). K_f_ is the pseudo-first-order rate constant (min^−1^). By plotting log (q_e_ − qt) versus (t), which results in the straight line illustrated in Figure 9A and Table 11, it is possible to obtain the values of q_e_ (mg/g) from the intercept and k_f_ (min^−1^) from the slope.

#### 3.5.2. Kinetic Second-Order Model

Equation (9) expresses the linear form of the pseudo-second-order equation:

t/q_t_ = 1/k_s_q_e_^2^ + t/q_e_ (9)

where the amounts of solute adsorbed (mg/g) at equilibrium and at time t are, respectively, q_e_ and q_t_ (min). K_s_ is the pseudo-second order rate equilibrium constant (g/mg. min). Plotting t/q_t_ versus (t) results in a straight line, which is illustrated in Figure 9B and Table 11. From the straight line, it is feasible to infer the value of K_s_ from the intercept and the value of q_e_ (mg/g) from the slope. Table 11 demonstrates that the correlation coefficient for the pseudo-second order kinetic model for AC(C-3) was greater than that of the pseudo-first order kinetic model (R^2^ = 0.98 and 0.99 for Fe(III) and Mn(II)), and the q_e_ values were very similar to the experimental values. Table 11 demonstrates that for the pseudo-second order kinetic model, the q_e_ values are quite similar to the experimental values and the correlation coefficient. When compared to the Fe(III) and Mn(II) pseudo-first-order equations, the pseudo-second-order equation of the experimental data performs more accurately, as seen by the high correlation coefficient value. This might lead us to conclude that chemisorption is an adsorption process in nature. This implies that the adsorption complies precisely with the pseudo-second order reaction, and the chemisorption mechanism appeared to control the adsorption of Fe(III) and Mn (II).

### 3.6. Thermodynamic Parameters

The feasibility and spontaneity of the biosorption process are represented by the thermodynamic parameters. Gibbs free energy, enthalpy changes, and entropy variations are typically employed to assess whether the mechanism of bio-sorption occurs spontaneously or not. The plot of ln k_d_ vs. 1/T at 298, 308, 323, and 338 K was used to analyze the typical variations of Gibbs free energy (ΔG°), enthalpy (ΔH°), and entropy (ΔS°), with the results provided in Table 12 [41,42].

The negative values of ΔG° at various temperatures in Table 12, showed that Fe(III) and Mn(II) spontaneously adsorbed onto AC(C-3) and that AC(C-3) had a higher affinity at higher temperatures. The endothermic nature of this biosorption process was revealed by the positive values of ΔH° for Fe(III) and Mn(II) for AC(C-3), respectively 19.31, 20.57 kJ/mol.). Furthermore, the enhanced entropy at the solid/solution interface was shown by the positive value of ΔS°. As the temperature rose, the free energy (ΔG°) decreased, indicating that the biosorption process was endothermic.

### 3.7. Application Study

The use of AC as an adsorbent for the treatment of groundwater was investigated. It can be seen from Table 13, that AC demonstrated a successful ability to handle Fe(III) and Mn(II) in groundwater. The capacity of AC to extract Mn(II) ions is usually greater than that of Fe(III) ions. In addition, the results showed that the percentage of Fe(III) and Mn(II) ions extracted was 69.4 and 76.4%, respectively. Thus AC(C-3) showed a good ability to handle groundwater Fe(III) and Mn(II) ions.

### 3.8. Comparison Study

To demonstrate the efficacy of AC(C-3) as an effective adsorbent for Fe(III) and Mn(II), it is necessary to compare its adsorption capacity to that of other known adsorbents. Table 14 compares the q_max_ values for Fe(III) and Mn(II) adsorption on different adsorbents to those of our adsorbents.

## 4. Conclusions

Activated carbon (AC) was prepared from rice husk from an agricultural byproduct. Rice husk was converted into activated carbon (AC) in an electric furnace at 700 °C. as it is chemically activated using three distinct processes: NaOH AC(C), acetic acid AC(C-1), and phosphoric acid AC(C-2), one sample by carbonization only AC(C-3) without chemical activation. The activated carbon effectively removed Fe(III) and Mn(II) from aqueous solutions. Carbon AC(C-3) produced the best results. AC(C-3) was found to be superior in terms of Mn(II) removal percentage and cost. Therefore, AC(C-3) was selected for further investigation. The sorption capacity was heavily influenced by the concentrations of Fe(III) and Mn(II) in the solution, as well as the pH value, contact time, adsorbent dosage, interfering ions, and temperature. The optimal pH for adsorption of Fe(III) and Mn(II) ions from aqueous solutions was determined to be pH 3 and pH 6, respectively. The optimal contact time for achieving equilibrium was determined to be 1 h for both Fe(III) and Mn(II) ions. The adsorption and kinetic models of Fe(III) and Mn(II) ions onto activated carbon (C-3) were fitted using the Langmuir adsorption and pseudo-second-order rate equations. The thermodynamic factors were calculated, and the reaction was established as being an endothermic mechanism. The activated carbon (AC(C-3)) demonstrated the best results for use as an adsorbent in water treatment applications to remove Fe(III) and Mn(II) ions from contaminated water.

## Figures and Tables

**Table 1:** The various types of the prepared activated carbon.

Symbols	Conditions	Methods
AC(C)	700 °C, 120 min	Activation by NaOH (1M)
AC(C-1)	700 °C, 120 min	Activation by (1M) H_3_PO_4_
AC(C-2)	700 °C, 120 min	Activation by (1M) CH_3_COOH
AC(C-3)	700 °C, 120 min	Carbonization

**Table 2:** Preliminary test for selection of adsorbent samples (C_o_ = Fe(III) and Mn(II) = 50 mg/L, pH= 1.5, 3, and 6 respectively, time = 2 h, wt. = 0.05 g, agitation speed = 100 rpm and temp. 25 °C). C_e_ is the concentration of Fe(III) and Mn(II) at equilibrium.

Type of Carbon	pH= 1.5				pH = 3				pH = 6	
	Fe(III)		Mn(II)		Fe(III)		Mn(II)		Mn(II)	
	Ce	% Removal	Ce	% Removal	Ce	% Removal	Ce	% Removal	Ce	% Removal
AC(C)	45.15	9.7	17.5	65	29.19	41.60	25.09	49.80	12.79	74.41
AC (C-1)	40.3	19.4	19.75	60.5	7.01	85.98	24.78	50.44	11.20	77.59
AC(C-2)	45.35	9.3	18.45	63.1	36.42	27.14	27.07	45.85	9.59	80.81
AC(C-3)	45.25	9.5	14.2	71.6	28.3	43.39	26.22	47.55	3.52	92.94

**Table 3:** The physical properties of the selected prepared activated carbon samples.

Sample	Apparent Density (g/cm^3^)	Packed Density (g/cm^3^)	Yield, %	Surface Area (m^2^/g)	Average Pore Radius nm	Total Pore Volume cc/g
AC(C-3)	0.477	0.591	17.6	228.46	1.845	0.2108
AC(C-2)	0.52	0.55	30.7	226.63	1.849	0.2095
AC(C-1)	0.48	0.64	24	69.89	2.848	0.0995
AC(C)	0.64	0.72	25.7	48.68	2.78	0.0677

**Table 4:** Elemental analyses of selected activated carbons.

Adsorbent,%	C	Al	O	Si	Cu	Zn	K	Cl	Ca	P	Mg	Na
AC(C-3)	36.12	−	33.51	16.46	2.96	2.44	0.52	−	−	7.99	-	-
AC(C-2)	43.5	0.06	41.72	14.18	0.06	-	0.22	0.05	0.09	-	0.07	0.04
AC(C-1)	52.12	0.04	51	10.95	0.04	-	0.67	0.02	-	5.75	0.11	0.07
AC(C)	48.86	0.03	39.42	6.24	0.01	-	0.58	0.13	0.2	-	0.15	5.21

**Table 5:** The influence of pH on the absorption and removal by AC(C-3) of Fe(III) and Mn(II) from aqueous solutions (conc. = 50 mg/L, time = 2 h, wt. = 0.05 gm, agitation velocity = 100 rpm and temp. 25 °C) by percentage.

Fe(III)			Mn(II)		
pH	Removal, %	Up Take, mg/g	pH	Removal, %	Uptake, mg/g
1	6.36	1.60	1.5	43.14	10.79
1.5	9.50	2.38	3	47.6	11.9
2	9.95	2.49	4	47.83	11.96
2.5	33.13	8.28	5	55.97	13.99
3	43.40	10.85	6	80.96	20.24

**Table 6:** The influence of contact time on the absorption and removal by AC(C-3) of Fe(III) and Mn(II) from aqueous solutions (conc. = 50 mg/L, time = 24 h, wt. = 0.05 gm, agitation velocity = 100 rpm and temp. 25 °C) by percentage.

Adsorbent	Contact Time	Fe(III)		Mn(II)	
		Removal, %	Uptake, mg/g	Removal, %	Uptake, mg/g
AC(C-3)	5 min.	31.21	7.8025	57.19	14.2962
	15 min	39.57	9.8933	57.95	14.4883
	30 min	49.63	12.4077	58.89	14.7227
	40 min	57.9	13.885	62.4	16.081
	50 min	57.79	13.972	62.95	16.236
	60 min.	61.58	15.3945	66.31	16.5772
	120 min.	60.56	15.14	65.8	16.449
	180 min.	60.62	15.154	66.08	16.5199
	24 h	60.16	15.0395	65.58	16.394

**Table 7:** The influence of adsorbent dosage on the absorption and removal by AC(C-3) of Fe(III) and Mn(II) from aqueous solutions (conc. = 50 mg/L, time = 60 min, wt. = 0.05 g, agitation velocity = 100 rpm and temp. 25 °C) by percentage.

Adsorbent	Adsorbent Dosage, g	Fe(III)		Mn(II)	
		Removal, %	Uptake, mg/g	Removal, %	Uptake, mg/g
AC(C-3)	0.02	48.22	24.11	58.7	20.35
	0.05	61.58	15.39	62.54	15.63
	0.075	67.58	11.26	71.86	12.91
	0.1	68.96	8.62	74.3	9.28
	0.2	69.72	4.35	75.5	4.71
	0.5	69.88	1.74	76.34	1.9

**Table 8:** The influence of initial concentration of metal ions on the absorption and removal by AC (C- 3) of Fe(III) and Mn(II) from aqueous solutions (conc. = 50 mg/L, time = 1 h, wt. = 0.05 gm, agitation velocity = 100 rpm and temp. 25 °C) by percentage.

Adsorbent	Initial Metal Ion Conc., (mg/L)	Fe(III)		Mn(II)	
		Removal, %	Uptake, mg/g	Removal, %	Uptake, mg/g
AC(C-3)	10	79.00	3.95	97.85	4.8925
	25	66.72	8.34	94.36	11.795
	50	43.46	10.865	92.30	23.075
	100	40.89	20.445	87.55	43.775
	200	29.25	29.25	71.99	71.997
	400	14.25	28.5	35.79	71.585

**Table 9:** Effect of interfering ions on Fe(III) and Mn(II) percent removal as well as uptake on AC(C-3) (from solutions) (contact time 1 h., pH = 3 for Fe(III) and pH = 6 for Mn(II), conc. = 50 mg/L).

Adsorbent	Metal Ions	% Removal of Metal	Up Take mg/g	Type of Ions		% Removal In Presence Interfering Ions	Uptake (qe) mg/g
AC(C-3)	Fe(III)	85.48	21.37	Cations in presence of Fe(III)	Fe^3+^	61.4	15.35
					Na^+^	38.4	9.6
					Mg^2+^	24.6	6.15
					K^+^	17	4.25
				Anions in presence of Fe(III)	Fe^3+^	46.4	11.6
					Cl-	33.2	8.3
					(SO_4_)^2−^	39.8	9.95
					(NO_3_)^−^	27	6.75
	Mn(II)	91.52	22.88	Cations in presence of Mn(II)	Mn^2+^	67.4	16.85
					Na+	33.8	8.45
					Mg^2+^	20.4	5.1
					k+	14.8	3.7
				Anions in presence of Mn(II)	Mn^2+^	53	13.25
					Cl-	40.2	10.05
					(SO_4_)_2−_	39.2	9.8
					(NO_3_)^−^	32.8	8.2

**Table 10:** Parameters of Langmuir and Freundlich for Fe(III) and Mn(II) adsorption using AC(C-3), respectively.

Adsorbent			Langmuir Isotherm			Freundlich Isotherm			Temkin Isotherm			
	Metal Ions	q_max_ (cal) (mg/g)	q_max_ (exp) (mg/g)	B	R^2^	n	K_f_ (mg/g)	R^2^	A_T_ (L/mg)	B_T_	b_T_	R^2^
AC(C-3)	Fe(III)	28.9	29.2	0.04	0.98	2.42	3.17	0.94	0.934	3.4	728.6	0.88
	Mn(II)	73.47	71.9	0.17	0.99	2.64	11.3	0.91	3.962	11.07	223.8	0.90

**Table 11:** Kinetic parameters for adsorption of Fe(III) and Mn(II) using AC(C-3), respectively.

Adsorbent		q_exp_	Pseudo-First Order			Pseudo-Second Order		
			K_f_ (min^−1^)	q_e_ calc. (mg/g)	R^2^	Ks (g/mg.h)	q_e_ calc. (mg/g)	R^2^
AC(C-3)	Fe(III)	15.39	−0.330	27.52	0.931	0.0067	16.9	0.98
	Mn(II)	16.75	−0.108	37.21	0.81844	0.0265	16.57	0.99

**Table 12:** Thermodynamic parameters for adsorption of Fe(III) and Mn(II) using AC(C-3), respectively.

Adsorbent		R^2^	ΔH° (KJ /mol)	ΔS° (J /mol. K)	∆G° (KJ /mol)			
					25 °C	35 °C	50 °C	65 °C
AC (C-3)	Fe(III)	0.92	19.31	120.96	−16.73	−17.94	−20.36	−21.57
	Mn(II)	0.85	20.57	125.94	−16.85	−18.10	−20.61	−21.87

**Table 13:** Treatment of Fe(III) and Mn(II) in ground water by using AC(C-3) adsorbent.

	Tested Parameters	Sample before Adding AC	Sample after Adding AC(C-3)
Physical parameters	Color	Colorless	Colorless
	Taste	Accp.	Accp.
	Odor	Odorless	Odorless
	Conductivity	986	920
	TDS	631	552
	Turbidity	2.96	0.59
	pH	7.23	7.21
	Chloride	151	143.8
	Sulfate	90	88
	Fe	0.95	0.290
	Mn	0.5	0.118
	Residual Aluminum	N.D	N.D
	Copper	N.D	N.D
	Zinc	N.D	N.D
	Nitrite	N.D	N.D
	Floride	N.D	N.D
	Total P.	N.D	N.D
	Free. Chlorine	1.3	1.1

**Table 14:** A comparison between the adsorption capacities for CIP and LEV with other sorbents in previous work.

Adsorbent	Adsorption Capacity (mg/g)		Reference
	Fe(III)	Mn(II)	
KOH activated silver (Ag) nanoparticle modified RH (AgNP-KOH-RH)	9.46	1.29	[43]
Hybrid chitosan-derived mesoporous spongy carbon (HCMSC) bio-adsorbent	165	-	[44]
Acid-activated kaolinite clay (AAC)	3.957	0.783	[45]
Titanium (IV) oxide (TiO_2_) nanoparticles supported on the AAC (TiO_2_–AAC)	3.989	0.678	[45]
Zeolite-4A	150.1	94.1	[46]
TiO_2_@Zeolites-4A nanocomposite	150.1	94.1	[46]
Biochar derived from the carbonization of palm kernel cake modified with KMnO_4_ and HNO_3_	70.67	-	[47]
Carbonized activated rice husk 700 °C AC(C-3)	28.9	73.47	Current study

## Data Availability

Data on the compounds are available from the authors.

## Sample Availability

Samples of the compounds are available from the authors.
